# Optimization of anaerobic soil disinfestation against Verticillium wilt in strawberry cultivation using local agrowastes as amendments

**DOI:** 10.3389/fpls.2024.1416401

**Published:** 2024-07-01

**Authors:** Paloma Hernández-Muñiz, Celia Borrero, Nieves Capote, Manuel Avilés

**Affiliations:** ^1^ Departamento de Agronomía, Escuela Técnica Superior de Ingeniería Agronómica (ETSIA), Universidad de Sevilla, Seville, Spain; ^2^ Área de Protección Vegetal Sostenible, Instituto Andaluz de Investigación y Formación Agraria, Pesquera, Alimentaria y de la Producción Ecológica (IFAPA) Centro Las Torres, Seville, Spain

**Keywords:** *Verticillium dahliae*, ASD, rice bran, residual strawberry extrudate, disease severity

## Abstract

The study explores anaerobic soil disinfection as an alternative to soil fumigants for controlling Verticillium wilt in strawberry crops. For this purpose, two agrowastes close to the strawberry-growing areas of Huelva province were tested as potential amendments for the control of Verticillium wilt: rice bran and residual strawberry extrudate. Furthermore, two application rates were evaluated: 13.50 and 20.00 t/ha for the rice bran and 16.89 and 25.02 t/ha for residual strawberry extrudate. Amended and anaerobically disinfested soils were compared with a non-amended soil under anaerobic conditions, a soil treated with the chemical fungicide metam sodium and an untreated soil. One week before the start of disinfection treatment, these soils were artificially inoculated with 250 microsclerotia/g dry soil of *Verticillium dahliae*. After disinfestation treatments, pathogens were quantified, and strawberry plants were cropped in a growth chamber to further evaluate Verticillium wilt severity, which was measured with a symptom scale in the same potting soils. Measurements of the anaerobic condition, pH and microbial population densities were performed, and the results showed significant differences between the different amendments. In addition, the treatment with rice bran at 20 t/ha recorded the lowest population density of *V. dahliae*. Likewise, it was possible to achieve a reduction in foliar disease severity in all amended treatments in similar percentage to those obtained by chemical treatment. These results suggest potential application of this technique for the control of Verticillium wilt in the strawberry-growing area of Huelva, reducing the use of chemical fumigants.

## 1 Introduction

Strawberry (*Fragaria x ananassa* Duch.) is a widely distributed crop with susceptibility to various soil pathogens, such as *Fusarium oxysporum* f.sp. *fragariae* (*Fof*) (Winks & Williams, 1965), *Macrophomina phaseolina* (Tassi) Goid, *Phytophothora cactorum* (Leb. & Cohn) J. Schrot., and *Verticillium dahliae* Kleb., the causal agent of Verticillium wilt. In Huelva, the strawberry production area in Spain, the management of these soilborne pathogens has become especially difficult since the entry into force of the current European legislation. This regulation prohibits the use of the most used soil fumigants, including 1,3-dichloropropene or chloropicrin. Dazomet and metam sodium are allowed one application every 3 years in the same field, subject to authorization (Regulation (EC) No. 1107/2009). The limited availability of tools to combat these diseases could cause great economic losses, so it is essential to look for more ecologically sustainable alternatives for the control of soil-borne pathogens.

Anaerobic soil disinfestation (ASD) is a promising alternative to the use of chemical fumigants for soilborne diseases management (Butler et al., 2012a; Shrestha et al., 2016; Márquez-Caro et al., 2022; Hernández-Muñiz et al., 2023). This technique consists of applying an amendment rich in labile carbon to the soil, watering to field capacity and then covering the soil with a plastic cover. Labile carbon stimulates the growth of micro-organisms, which consume oxygen, and the plastic mulching acts as a barrier to gas exchange, thus establishing anaerobic conditions (Momma et al., 2013; Poret-Peterson et al., 2019). Depending on the carbon source used, different microbial communities will be generated under such conditions (Shennan et al., 2014). Microbial communities play a crucial role in the success of pathogen disinfestation, making the selection of the appropriate amendment an important decision for the effectiveness of ASD treatment (Hewavitharana & Mazzola, 2016; Liu et al., 2016; Poret-Peterson et al., 2019). The mechanisms of pathogen suppression with ASD are not still clearly understood, but the production of organic acids produced by the anaerobic decomposition of the added carbon, the release of volatile compounds and the resulting biocontrol micro-organisms after ASD seem to be involved (Poret-Peterson et al., 2019.; Shennan et al., 2014). Recently, Littrell et al. (2024) proved the importance of volatile fatty acids (VFAs) in reducing the viability of *Fusarium oxysporum*, with sandy soils being more suppressive to the disease than clay soils at the same concentration of VFAs. This technique is widely used in strawberry crop to manage soilborne diseases like Verticillium wilt (Goud et al., 2004; Shennan et al., 2018). Management of Verticillium wilt is very important for the economic viability of the crop due to the rapid progress of the disease, leading to plant death and severe yield losses. Microsclerotia of this pathogen can persist in the soil over years (Wilhelm, 1955; Green, 1980), so that soil disinfestation techniques are required. For the control of Verticillium wilt on strawberry crops, temperatures above 22.5°C (Ebihara & Uematsu, 2014) and an accumulation of 50,000 mVh below 200 mV are necessary (Shennan et al., 2018). One of the most used carbon sources in ASD is rice bran. This amendment has a high labile carbon and has been successfully used for the management of various soil pathogens in ASD treatment (Serrano-Pérez et al., 2017; Mazzola et al., 2018; Shennan et al., 2018; Hernández-Muñiz et al., 2023). Another successful by-product recently tested against *M. phaseolina* (Márquez-Caro et al., 2022) and *F. oxysporum* f.sp *fragariae* (Hernández-Muñiz et al., 2023) is the residual strawberry extrudate. This by-product has a similar concentration of labile carbon as rice bran and could be used as an amendment for ASD against Verticillium wilt. Both wastes can be found in industries close to the strawberry-growing area of Huelva.

The main objective of the present study was to optimize the ASD strategy in Spain for reducing *V. dahliae* microsclerotia in soil and alleviate the severity of symptoms caused by *V. dahliae* on strawberry crops in Spain to economically sustainable levels using by-products from industries near the strawberry crop area of Huelva, Spain.

## 2 Materials and methods

The effect of ASD on the reduction of *V. dahliae* microsclerotia and the severity of Verticillium wilt was evaluated. For this purpose, a common sandy soil extracted from a strawberry plantation (37.398045, -7.075881) in the growing area of Huelva was used to carry out the experiment under conditions similar to those in the field. The amendments used were rice bran as a standard amendment and residual strawberry extrudate at different amendment doses for optimization: 13.5 and 20 t/ha for rice bran and 16.89 and 25.02 t/ha for residual strawberry extrudate. One week before the addition of the amendments (start of the treatments), the soil was artificially inoculated with *V. dahliae*. At the end of the treatments, a trial was carried out under controlled climatic conditions in a growth chamber, where strawberries were grown in the soil resulting from the treatments (ASD, control and chemical) to evaluate the disease caused by *V. dahliae*.

### 2.1 Soil and amendment characterization

Before starting the trials, a physico-chemical characterization of the soil and the amendments used was carried out.

#### 2.1.1 Soil characterization

Three soil samples (without previous disease) were collected from the first 20 cm, excluding the upper layer. Soil characterization was determined by the Agricultural Research Service of the University of Seville (**Table 1**).

**Table 1:** Physicochemical parameters of the soil used in experiment.

Parameters	Values^+^
**pH (extractor ½, 5 p/V)**	5.52 ± 0.06
**Electrical conductivity (µS/cm) (extractor 1/5 p/V)**	216.20 ± 13.27
**Olsen P (mg/Kg)**	62.18 ± 1.75
**Oxidable organic matter (%)**	0.42 ± 0.01
**Oxidable organic carbon (%)**	0.24 ± 0.00
**Total N (%)**	0.03 ± 0.01
Exchange cations (soluble in ammonium acetate 1N pH 7)	
**Ca (cmolc/Kg)**	1.17 ± 0.09
**Mg (cmolc/Kg)**	0.37 ± 0.02
**K (cmolc/Kg)**	0.52 ± 0.03
**Na (cmolc/Kg)**	0.18 ± 0.01
Available trace elements (soluble in DTPA-TEA-CaCl_2_)	
**Fe (mg/Kg)**	49.92 ± 1.25
**Mn (mg/Kg)**	11.33 ± 0.53
**Zn (mg/Kg)**	4.23 ± 0.12
**Cu (mg/Kg)**	2.73 ± 0.03
Texture:	
**Silt (%)**	7.18 ± 0.08
**Clay (%)**	3.59 ± 0.10
**Sand (%)**	89.24 ± 0.06

^+^Data represent mean ± standard error.

#### 2.1.2 Amendment characterization

Rice bran (Arrozúa, Seville, Spain) and residual strawberry extrudate (Svz, Huelva, Spain) were employed as amendments. Total carbon, nitrogen and sulphur were determined by the Agricultural Research Service of the University of Seville using LECO CNS-Trumac Elemental Autoanalyzer (Michigan, USA) (**Table 2**).

**Table 2:** Determination of oxidable and total organic carbon, total nitrogen, total sulphur, and the humidity of used amendments.

Amendment	Oxidable organic carbon (%)	Total C (%)	Total N (%)	Total S (%)	Humidity (%)
**Rice bran**	46.51	50.19	2.21	0.24	4.51
**Residual strawberry extrudate**	37.18	52.37	1.95	0.18	5.42

### 2.2 Evaluation of *V. dahliae* microsclerotia reduction in soil

#### 2.2.1 Soil inoculum preparation

*Verticillium dahliae* strain V.31 was used to prepare the soil inoculum. V.31 was isolated from strawberry plants and is conserved in the Plant Pathology Laboratory of the University of Seville. The isolate was grown on PDA medium for one week. Then, rice was inoculated in polypropylene bags with filters (PPD75/REH/V37–53, SacO2, Deinze, Belgium) according to the procedure described by Benson and Parker (2015). Once the pathogen had completely colonized the rice, it was dried, ground, and mixed with the soil according to Hernández-Muñiz et al. (2023). After one week, inoculum-soil titration was performed to estimate the final microsclerotia concentration according to Harris et al. (1993). The estimated soil inoculum concentration was 2.42×10^7^ microsclerotia/g soil.

#### 2.2.2 Experimental design

Two trials including seven treatments and three repetitions for each were performed. Soils were inoculated with 250 microsclerotia/g dry soil, homogenized with 4.1 Kg of soil with an automatic homogenizer (Heidolph Reax 20, Schwabach, Germany) and then placed in a black bag within a 2.5L internal volume container (experimental unit). The containers were incubated during one week at room temperature for the correct establishment of the pathogen. The number of *V. dahliae* microsclerotia was re-estimated on PDA medium to check the good establishment of the pathogen into the soil following the procedure described by Harris et al. (1993). The average density of microsclerotia recorded was 25 microsclerotia/g dry soil. Amendments and corresponding water to each treatment were added and homogenized with an automatic homogenizer. The amendment doses used for rice bran were 13.50 and 20.00 t/ha (Mazzola et al., 2018; Márquez-Caro et al., 2022; Hernández-Muñiz et al., 2023). and 16.89 and 25.02 t/ha for residual strawberry extrudate (Márquez-Caro et al., 2022.; Hernández-Muñiz et al., 2023). The volume of water needed to reach field capacity was calculated considering the soil and amendments humidity. Then, bags were immediately closed. To facilitate redox measurements during the trial, 15 mL falcon tubes were placed with one end cut off (in contact with the soil) and the other end (outside the container) closed with a stopper. In addition, an unamended treatment under anaerobic conditions, an unamended treatment under aerobic conditions (control treatment) and a chemical treatment with 50% (w/v) metam sodium at 300 l/ha (Raisan-50, Lainco S.A., Barcelona, Spain) were included. Anaerobic treatments had a duration of 25 days and the fumigant treatment lasted for 15 days, as recommended in the product label.

The trials were placed randomly in a growth chamber and incubated in dark at 33/23°C. The incubation temperature of the treatments was chosen according to temperature in Huelva in the summer months to simulate real field conditions.

#### 2.2.3 Soil redox and pH

During treatments, several redox measurements were conducted at different times following the methodology outlined by Hernández-Muñiz et al. (2023). The SensoLab Benchtop pH/ORP Meter (PM1000) with the ORP1000 Polycarbonate Laboratory ORP Sensor (Sensorex Corporation, California, USA) was used for redox measurements. Soil redox potential values were corrected to be relative to the redox potential of a standard hydrogen electrode. For modification of the ORP reading in mV to Eh mV, the addition of 201 mV was necessary (Fiedler et al., 2007). The critical redox potential of soil, indicative of reduced conditions (Rabenhorst & Castenson, 2005), was calculated using the formula [CEh= 595mV – 60 mV (soil pH)] was used. The value of 264 mV was determined as the threshold below which soil is considered as anaerobic at a soil pH of 5.52 (Shennan et al., 2018). Soil pH was determined with the GLP22 Crison pH meter (Hach Lange Spain, S.L.U, Barcelona, Spain) after ASD treatments following Hernández-Muñiz et al. (2023).

#### 2.2.4 Potential biocontrol microbes

Microbial density of usual biocontrol agents, including *Trichoderma* spp., fluorescent *Pseudomonas* spp. and copiotrophic bacteria, was determined in soils by dilution plating using a combination of selective culture media according to Borrero et al. (2004). *Streptomyces* spp. density was determined using 1/50 tryptone soybean medium as described by Mazzola et al. (2018). Samples were taken from each repetition after treatments and following one week of aeration.

#### 2.2.5 Quantification of *V. dahliae*

*V. dahliae* DNA was extracted in triplicated from each repetition of aerated soil sample (experimental unit) using DNeasy PowerSoil Pro kit (QIAGEN, Germany) according to the manufacturer’s procedure. DNA was quantified in triplicate in a Nanodrop spectrophotometer (Thermo Fisher Scientific, Waltham, MA, USA). Real-time polymerase chain reaction (qPCR) for the detection and quantification of *V. dahliae* was carried out following the conditions described by Bilodeau et al. (2012) with some modifcations: qPCR reactions were performed in 96-well plates using a CFX Connect thermocycler (Bio-Rad) in a 20 μL final volume. This contained 1x SensiMix (SensiMixTM Probe Kit, Bioline), 0.1 mg/mL BSA, 1 μM each primer (Bilodeau et al., 2012), 5 mM TaqMan probe marked with 6′FAM fluorescein (Bilodeau et al., 2012), and 5 μL of extracted DNA. To discard false-negative amplifications, an internal positive control (IPC) was used consisting of lambda (λ) bacteriophage DNA (1 pg) added to each sample and amplified in duplex qPCR assays using primers λ -F (5´-GGT GGA AAC CGC ATT CTG TAC-3´) and λ-R (5´-CCG TCG AGA ATA CTG GCA ATT T-3´) and a HEX-TaqMan probe (5’-TCG TGCT GTC GCG GAT CGC AGG T-3’). Amplifications were carried out at 95°C for 10 minutes, and 55 cycles of 15 s at 95°C and 30 s at 62°C. In each run, sterile distilled water was used as a negative control. A standard curve was constructed by serial dilutions of *V. dahliae* V.31 genomic DNA included in each qPCR assay in triplicate. Quantifications were calculated as pg of *V. dahliae* DNA per g of soil using the CFX Connect software (Bio-Rad).

### 2.3 Assessment of disease severity after soil treatments

#### 2.3.1 Experimental design

After treatment, soil samples were aerated for one week. Subsequently, the soil samples from each repetition were transferred into three 0.65L pots (n= 9 pots per treatment and 3 pots per block). One bare root strawberry plant of the ‘Splendor’ variety (Plant Sciences-Berry Genetics, Watsonville, California) was transplanted into each pot. Plants were randomized within each block in a growth chamber and maintained for 235 days at 25°C day and 22°C night with a 12-h photoperiod. The final experimental design encompassed 2 trials× 7 treatments × 3 blocks × 3 repetitions.

#### 2.3.2 Disease development

Weekly observations were conducted to monitor the progression of the disease caused by *V. dahliae*. Disease foliar severity was assessed considering leaf wilting and dwarfing. Measurements were made by counting affected and total leaves and the proportion between them ranged from 0 (healthy plant) to 1 (totally affected plant). At the end of the plant trials, root severity was measured as percentage of affected roots (necrotic roots): 0 = all roots were healthy and 1 = all roots were necrotic. To confirm the presence of *V. dahliae*, crown piece samples were disinfected by immersion in 1% sodium hypochlorite for two minutes, followed by a two-minute water rinse and drying under sterile conditions for two hours. Finally, the crown pieces were placed on Verticillium selective medium (Papavizas and Klag, 1975).

#### 2.3.3 Potential biocontrol microbes in rhizosphere

The microbiological biocontrol populations were determined in the strawberry rhizosphere as described in section 2.2.4. For this process, ten grams of rhizosphere soil were collected from a mixture of the rhizospheres of the three repetitions within each block and treatment.

### 2.4 Statistical analysis

Data collected from two trials were analyzed with the software Statgraphics Centurion 18 (18.1.13 version; Statgraphics Technologies, Inc., The Plains, VA). Data coming from the two trials were pooled for statistical analysis after finding no significant disinfestation treatment x trial interaction in factorial ANOVA. Disinfestation treatments, trials, and their interaction were treated as fixed effects, blocks nested in trial and their interaction were considered as random effects. The variables analyzed were foliar disease severity and the percentage of necrotic roots (n=18); cumulated anaerobic conditions and pH in the soil post-disinfestation (n=6) and population density of the microbiological groups (*Trichoderma* spp., copiotrophic bacteria, *Streptomyces* spp. and fluorescent *Pseudomonas* spp.) present in the soil post-disinfestation and at the end of the plant trials (n=6). When necessary, data were transformed for compliance with ANOVA requirements and were tested using Levene, Bartlett and Cochran’s tests. Means were compared using Duncan’s test.

## 3 Results

### 3.1 Soil physicochemical parameters

All amended treatments showed higher cumulative anaerobic condition values, measured as Eh mVh below 264 mV, than the anaerobic unamended treatment. Likewise, the amended treatments showed no significant differences for the accumulation of anaerobic conditions among them (**Table 3**).

**Table 3:** Effect of disinfestation treatments on cumulative Eh mV h and pH.

Amendment	Dose (t/ha)	Cumulative Eh mV h below 264 mV^a^	pH^b^
**Rice bran**	13.50	62,716 ± 6,394 **ab**	6.8 ± 0.26 **b**
**Rice bran**	20.00	94,304 ± 13,899 **a**	7.29 ± 0.12 **a**
**Residual strawberry extrudate**	16.89	87,355 ± 8,802 **ab**	6.62 ± 0.11 **bc**
**Residual strawberry extrudate**	25.02	68,907 ± 9,620 **ab**	6.57 ± 0.08 **bcd**
**Anaerobic unamended**	–	46,309 ± 9,935 **c**	5.63 ± 0.15 **e**
**Aerobic unamended**	–	–	6.20 ± 0.04 **d**
**Metam sodium**	50% w/v 300 l/ha	–	6.43 ± 0.15 **cd**

Data represent the mean ± standard error (n=6). Values followed by different letters indicate significant differences according to ANOVA and Duncan test (*P*<0.05).

a Cumulative Eh mV h below 264 mV: measures were taken during the duration treatment. Data for analysis were transformed with X^0.3^.

b pH: measures were taken just after treatments.

Regarding pH, all amended treatments, except for a high dose of residual strawberry extrudate, showed higher values than the aerobic unamended. In addition, the high dose residual strawberry extrudate and metam sodium treatments showed no significant differences with the aerobic unamended. On the other hand, the high dose of rice bran showed the highest pH (**Table 3**).

### 3.2 Soil microbial parameters

#### 3.2.1 Quantification of *V. dahliae* after treatments

Significant differences were observed for the amount of *V. dahliae* DNA/g of soil among treatments. However, no differences were found between the doses tested within each amended treatment (**Table 4**). All treatments showed lower *V. dahliae* inoculum density than the aerobic unamended treatment. The inoculum density of *V. dahliae* after the metam sodium treatment did not significantly differ from that observed with residual strawberry extrudate treatments at the two assessed doses nor from the inoculum density following the anaerobic unamended treatment. Rice bran treatments recorded the lowest amount of *V. dahliae* DNA/g of soil (**Table 4**).

**Table 4:** qPCR quantification of *Verticillium dahliae* in soil after treatments.

Amendment	Dose (t/ha)	*V. dahliae* density (pg DNA/g soil (x10^-1^)^a^
**Rice bran**	13.50	2.60 ± 2.19 **de**
**Rice bran**	20.00	0.24 ± 0.17 **e**
**Residual strawberry extrudate**	16.89	15.9 ± 10.8 **bc**
**Residual strawberry extrudate**	25.02	24.2 ± 14.0 **b**
**Anaerobic unamended**	–	5.63 ± 3.23 **cd**
**Aerobic unamended**	–	380.0 ± 77.1 **a**
**Metam sodium**	50% w/v, 300 l/ha	4.57 ± 1.35 **bc**

Data represent the mean ± standard error (n = 6) Values followed by different letters indicate significant differences according to ANOVA and Duncan test (*P*<0.05).

a *V. dahliae* density (pg DNA per gram of soil (x10^-1^): Quantification of *V. dahliae* by qPCR was taken after one week of aeration of the treatments. Data for analysis were transformed with X^0.15^.

#### 3.2.2 Biocontrol microbes’ population densities

After treatments, rice bran amended with 20 t/ha showed high population density of *Trichoderma* spp. and copiotrophic bacteria. Likewise, the treatment amended with 13.5 t/ha of rice bran showed significant differences in copiotrophic bacteria populations compared to the rest of amended treatments, but not in the *Trichoderma* spp. populations (only significant differences with the aerobic amended and metam sodium treatments). The treatments with residual strawberry extrudate showed no differences with the metam sodium treatment and with the aerobic and anaerobic unamended treatments in the density of recorded *Trichoderma* spp. populations and copiotrophic bacteria (**Table 5**). No significant differences were observed among treatments in the population density of fluorescents *Pseudomonas* spp. and *Streptomyces* spp. (data not shown).

**Table 5:** Population densities of potential biocontrol microbes post treatments.

Amendment	Dose	*Trichoderma* spp. density^a^	Copiotrophic bacteria density^b^
	(t/ha)	CFU g^-1^ dry soil (x10^3^)	CFU g^-1^ dry soil (x10^7^)
**Rice bran**	13.50	16.2 ± 60.3 **b**	7.49 ± 0.83 **b**
**Rice bran**	20.00	50.2 ± 97.6 **a**	17.78 ± 3.02 **a**
**Residual strawberry extrudate**	16.89	4.51 ± 3.26 **bc**	1.36 ± 0.67 **c**
**Residual strawberry extrudate**	25.02	9.06 ± 2.69 **bc**	2.01 ± 0.91 **c**
**Anaerobic amended**	–	4.19 ± 0.72 **bc**	1.33 ± 0.95 **c**
**Aerobic amended**	–	3.57 ± 0.54 **c**	2.23 ± 0.96 **c**
**Metam sodium**	50% w/v, 300 l/ha	0 ± 0 **c**	1.33 ± 0.80 **c**

Data represent the mean ± standard error (n = 6). Values followed by different letters indicate significant differences according to ANOVA and Duncan test (*P*<0.05).

^a^Colony forming unit (CFU) of *Trichoderma* spp. post treatments after one week of aeration; ^b^CFU of copiotrophic bacteria post treatments after one week of aeration.

After the plant trial, no significant differences were observed between treatments for biocontrol microbes, *Trichoderma* spp., copiotrophic bacteria spp., fluorescent *Pseudomonas* spp., and *Streptomyces* spp. (data not shown).

### 3.3 Disease severity

**Figure 1:**
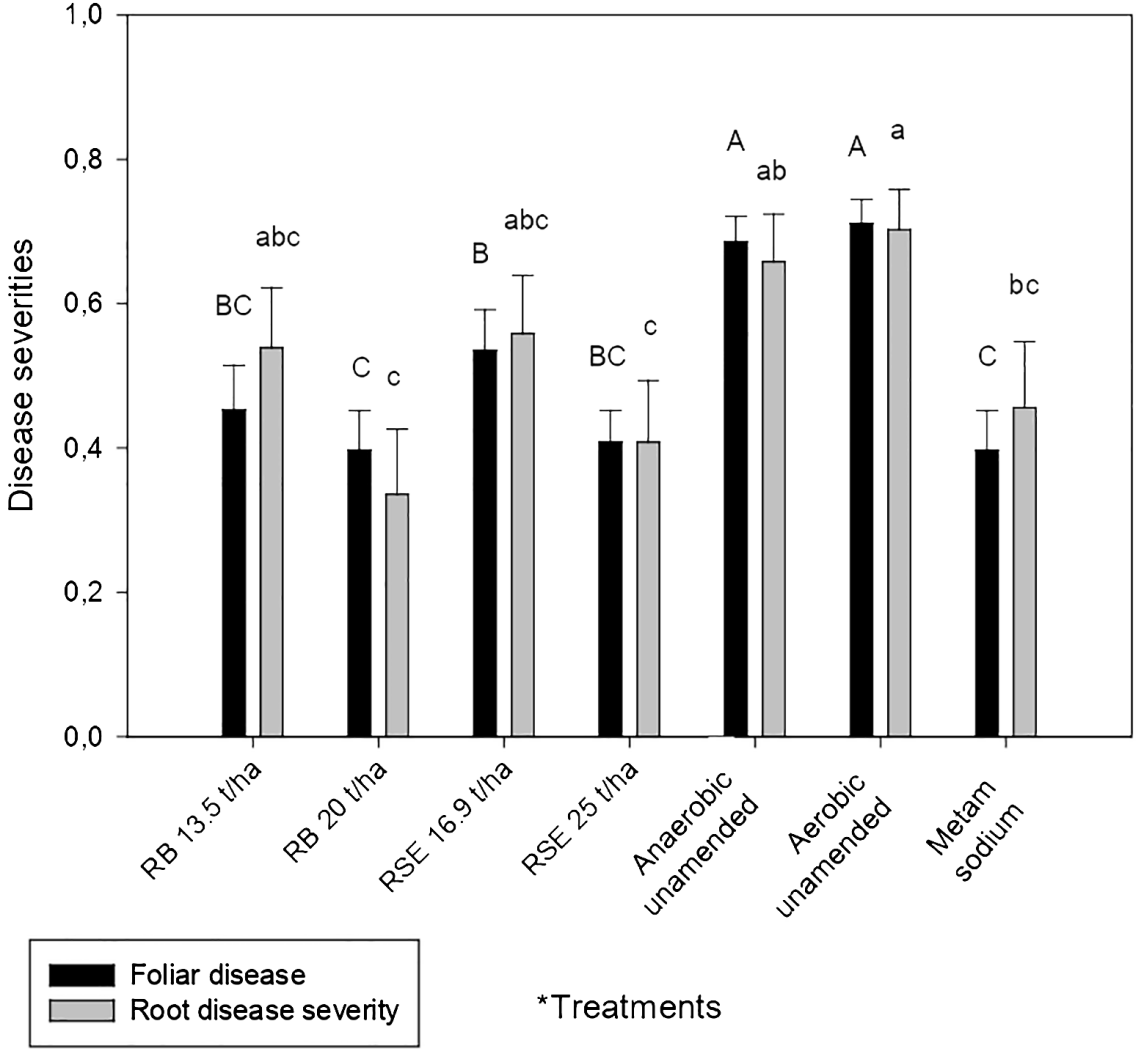
Effect of treatments on foliar and root disease severity (diseased tissue proportion) in strawberry. Columns represent the average values of foliar or root severity (n = 18) and the bars represent the standard error. Different letters above columns indicate significant differences according to ANOVA followed by the Ducan test (*P*<0.05). Data of root disease severity was transformed for statistical analysis as x^1.8^. *Treatments: RB, rice bran amendment; RSE, residual strawberry extrudate amendment; Anaerobic unamended; Aerobic unamended; Metam sodium.

Treatments including amendments and metam sodium exhibited lower foliar disease severity than the aerobic and the anaerobic unamended treatments. In addition, all amended treatments, except the amended with 16.89 t/ha of residual strawberry extrudate, showed similar severity values to the metam sodium treatment (**Figure 1**).

A reduction in root severity was only observed in comparison to the anaerobic and aerobic unamended soil when high doses of both amendments were applied. Metam sodium also decreased root severity compared to the aerobic unamended but not in comparison to the anaerobic unamended soil (**Figure 1**).

## 4 Discussion

In the present study, a reduction in disease severity was observed in the treatments with rice bran and residual strawberry extrudate at 20 and 25 t/ha respectively. In addition, rice bran at 20 t/ha reduced *V. dahliae* density compared to aerobic and anaerobic treatments without amendment. Additionally, the efficacy of these treatments were comparable to that achieved by metam sodium treatment. Notably, the 20 t/ha rice bran amendment achieved the greatest reduction in *V. dahliae* inoculum density.

The efficacy of this kind of residues as amendments in ASD to control soil pathogens is well-known. Studies conducted by Serrano-Pérez et al. (2017); Shennan et al. (2018), and Márquez-Caro et al. (2022) have demonstrated the ability to reduce the number of propagules of *Phytophthora nicotianae*, *V. dahliae*, and *M. phaseolina*, respectively, using rice bran. Furthermore, Márquez-Caro et al. (2022) and Hernández-Muñiz et al. (2023) observed a decrease in disease severity caused by *M. phaseolina* and *F. oxysporum* f. sp. *fragariae*, respectively, following treatments with rice bran (20 t/ha) and residual strawberry extrudate (25 t/ha and 60 days of anaerobiosis).

### 4.1 Influence of the anaerobic conditions

Shennan et al. (2018) proposed that a threshold of 50,000 mVh at 25°C is required to reduce 80–100% *V. dahliae* propagules. Despite treatments in this study were conducted at 33°C, only the amended treatments reached this threshold. Nevertheless, a reduction of *V. dahliae* inoculum density in soil was recorded in all treatments under anaerobic conditions, including the unamended treatment. However, only the amended treatments exhibited a reduction in foliar severity compared to the aerobic unamended treatment, an effect not observed in the unamended treatment under anaerobic conditions. This suggests that, besides the reduction of inoculum density of *V. dahliae* in soil, additional modifications were induced by using such by-products in soil amendment under anaerobic conditions.

### 4.2 Influence of soil pH

Several authors have reported changes in soil physico-chemical parameters following ASD treatments, including alterations in soil pH that were related to soil type, amendments used, anaerobic conditions and temperature (Rosskopf et al., 2015). Soil type is a crucial factor in the impact of ASD treatment on soil pH. Previous research has shown that soils rich in organic matter with finer-texture (clay soils) and alkaline pH have high buffering capacity so that the effect of ASD treatment on soil pH is limited (Bohn et al., 1985), and even the pH of alkaline soils can decrease due to the generation of organic acids and the accumulation of dissolved carbon dioxide (Inglett et al., 2005). In contrast, other studies have indicated that coarser-textured soils (sandy soils) have low buffering capacity so are more likely to show significant responses in soil pH to ASD treatment or organic matter additions (Butler et al., 2012a). Therefore, in studies related to ASD, it is common to find studies where authors reported a decrease in pH, attributed to the release of volatile acids (Momma et al., 2006; Muramoto et al., 2016), and others, where an increase in pH was reported after anaerobic treatments (Shrestha et al., 2021; Márquez-Caro et al., 2022; Hernández-Muñiz et al., 2023).

Previous studies have observed that decomposition of organic amendments can potentially raise soil pH by releasing basic cations (Marschner and Noble, 2000; Xu et al., 2006). This phenomenon may become the predominant process in the absence of significant organic acid formation during anaerobic decomposition. On the other hand, studies by Littrell et al. (2024) described the importance of volatile fatty acids in the suppression of *F. oxysporum* (Fo). Furthermore, they observed a higher suppression of the disease in sandy soils than in clay soils at the same concentration of added VFAs, which was attributed to the lower buffering capacity in sandy soils than in clay soils. Similarly, studies by Littrell et al. (2024) also observed that at higher soil pH, VFAs dissociation increases, reducing Fo suppression by VFAs. Therefore, they deduced that volatile fatty acids are not the only ones involved in disease suppression. Studies by Hernández-Muñiz et al. (2023) and Márquez-Caro et al. (2022) on sandy soils with a similar structure to the one used in this research, also detected an increase in pH in soils treated with ASD. In addition, Hernández-Muñiz et al. (2023) related the decrease of propagules and the severity of the disease caused by *F. oxysporum* f.sp. *fragariae* since it is well established that a basic soil pH reduces the severity of Fusarium wilt (Borrero et al., 2004).

Our study found that the ASD treatments resulted in an increase in soil pH. The treatment that was amended with rice bran at 20 t/ha had the highest pH value among all treatments. In addition, this treatment had the highest reduction of *V. dahliae* inoculum density in the soil and the lowest foliar severity among the amended treatments. However, it is important to note that *V. dahliae* usually thrives in alkaline soils with a pH between 6 and 9. Therefore, the reduction of propagules in our study observed in soils with higher pH indicates that the reduction of propagules and disease severity may be influenced by other variables such as soil texture, the rate of mineralization of organic matter and soil microbial activity (Gamliel et al., 2000).

### 4.3 Influence of certain soil microorganisms

The study quantified the populations of *Trichoderma* spp., copiotrophic bacteria, *Streptomyces* spp., and fluorescent *Pseudomonas* spp. after the treatments and plant trial. However, only significant differences in the populations of *Trichoderma* spp. and copiotrophic bacteria were found after the treatments, and no differences were observed after the plant trials. In both cases, the treatments amended with rice bran (both doses) showed an increase in these populations, with the high amended dose (20 t/ha) resulting in a higher population of these potential biocontrol agents. *Trichoderma* spp. and copiotrophic bacteria are known to help control of soil-borne diseases such as *M. phaseolina*, *F. solani* (Pastrana et al., 2016), and *V. dahliae* (Mercado-Blanco et al., 2004; Mirmajlessi et al., 2016). They are also suppressive soil indicators against diseases (Van Bruggen and Semenov, 1999; Kotsou et al., 2004). The population density of copiotrophic bacteria was higher in the treatments amended with rice bran, with a significant difference between the two doses. The higher dose of 20 t/ha of rice bran recorded a higher population density of copiotrophic bacteria. This could be due to the fact that rice bran provides more easily assimilated carbon compared to the residual strawberry extrudate (Cederlund et al., 2014). The higher pH in the treatment with 20 t/ha of rice bran may have favored the greater presence of microorganisms. Previous studies reported that copiotrophic bacteria compete for organic nutrients (Jones et al., 1993) and depend on a high pH (Woltz and Jones, 1981). Therefore, the higher population numbers of *Trichoderma* spp. and copiotrophic bacteria, aided by higher availability of labile carbon and a higher pH, could explain the lower inoculum density of *V. dahliae* in soil and the lower severity found in the rice bran amended treatments.

The addition of organic amendments to the soil can lead to changes in the populations of microorganisms present in the soil (De Corato, 2020). These populations can vary depending on the carbon source used (Butler et al., 2012b). Encouraging soil microbial communities could be an effective method to develop natural suppression of soil-borne plant pathogens, including *V. dahliae* (De Corato, 2021; Kowalska, 2021). Poret-Peterson et al. (2019) through their metagenomic analysis of soil after ASD treatments observed that microorganisms responsible for nitrogen fixation responded positively to ASD treatments. This increase in nitrogen-fixing microorganisms in ASD-treated soils has implications not only for crop health, but also for crop nutrition. The ability of these microorganisms to fix atmospheric nitrogen could potentially improve the availability of this essential plant nutrient. This phenomenon suggests a beneficial mechanism for optimizing the nutrition of ASD-treated crops, which could translate into improved yields and overall health of agricultural crops. However, further studies are required to fully understand the implications and practical applications of this phenomenon in agriculture.

### 4.4 Organic residue revaluation

One of the main targets for Spain is to implement environmental legislation (EIRSs) for the improvement of waste management and the development of the Circular Economy. The new Circular Economy Action Plan adopted in March 2020 is one of the main pillars of the European Green Deal. In addition, the new Common Agricultural Policy (CAP) also supports the transition towards sustainable agriculture. Among the objectives of this new policy are to reduce by 50% the use of pesticides and nutrient losses from fertilizers, while ensuring that soil fertility is not impaired, and to increase by at least 25% the areas devoted to organic farming (European Commission, 2022). Therefore, the use of rice bran and residual strawberry extrudate in anaerobic soil disinfestation in strawberry crop is proposed as a promising technique that contributes to the circular economy and meets CAP objectives. The addition of these residues and by-products to agricultural soil could be used as substitutes for chemical fumigants and fertilizers. By using agrowaste as soil amendment has a positive impact on the organic matter content and fertility of soil, thereby improving health (De Corato et al., 2024) and suppressiveness of the soil against various stress factors, including those caused by soil-borne plant pathogens (De Corato, 2023).This benefit could be utilized by farmers to decrease the necessity of adding chemical fertilizers to the soil, promoting the use of organic residues as fertilizers. Research conducted by Shennan et al. (2018) has shown that soils with higher levels of nitrogen or phosphorus have a lower incidence or higher suppressiveness of diseases such as Verticillium wilt in strawberries and potatoes. Additionally, Shennan et al. (2018) noted an increase in strawberry yield in the field with the addition of rice bran. Consequently, both organic residues could be used as organic fertilizers. This would improve crop productivity and soil suppressiveness, while also meeting European regulatory requirements. In addition, it would give added value to both the waste and the strawberry crop by making it eligible for organic farming.

## 5 Conclusion

The reduction of the inoculum density of *V. dahliae* in soil, achieved by the tested treatments, does not necessarily result in a reduction in the severity of Verticillium wilt of strawberry. Only the treatments that included organic amendments and metam sodium managed to reduce the severity compared to the control.

These residues can also be used as amendments for ASD in other horticultural crops. The pathogen *V. dahliae* is particularly problematic due to its wide host range and the issues that arise after the removal of methyl bromide and other soil fumigants used for soil disinfestation. Therefore, the ASD technique utilizing these residues could be a viable alternative for other crops.

## Data availability statement

The raw data supporting the conclusions of this article will be made available by the authors, without undue reservation.

## Author contributions

PH-M: Investigation, Methodology, Writing – original draft. CB: Conceptualization, Funding acquisition, Investigation, Methodology, Supervision, Visualization, Writing – review & editing, Resources, Validation. NC: Methodology, Resources, Supervision, Writing – review & editing. MA: Conceptualization, Data curation, Formal analysis, Funding acquisition, Investigation, Methodology, Project administration, Resources, Supervision, Validation, Visualization, Writing – review & editing.
